# Molecular Characteristics of First IMP-4-Producing *Enterobacter cloacae* Sequence Type 74 and 194 in Korea

**DOI:** 10.3389/fmicb.2017.02343

**Published:** 2017-11-28

**Authors:** Jong Ho Lee, Il Kwon Bae, Chae Hoon Lee, Seri Jeong

**Affiliations:** ^1^Department of Laboratory Medicine, Yeungnam University College of Medicine, Daegu, South Korea; ^2^Department of Dental Hygiene, College of Health and Welfare, Silla University, Busan, South Korea; ^3^Department of Laboratory Medicine, Kosin University College of Medicine, Busan, South Korea

**Keywords:** IMP-4, CMY, carbapenem, class 1 integron, *Enterobacter cloacae*

## Abstract

The worldwide dissemination of carbapenemase-producing *Enterobacteriaceae* (CPE) has become a major therapeutic concern in clinical settings. *Enterobacter cloacae* is a major pathogen that causes serious hospital-acquired infections. We investigated the clinical characteristics and molecular mechanisms of the first IMP-4-producing *E. cloacae* clinical isolates in Korea. Five carbapenemase-producing *E. cloacae* strains out of 792 *E. cloacae* clinical isolates, which have been identified at a university hospital in Korea between March 2014 and February 2016, were included in this study. Antimicrobial susceptibilities to imipenem, meropenem, and ertapenem were tested using *E*-test. Carbapenemase determinant screening, genetic environment, and multilocus sequence typing were conducted using PCR and sequencing analysis. All isolates were not susceptible to at least one of the tested carbapenems and presented highly similar pulsed-field gel electrophoresis (PFGE) patterns, evidencing hospital-wide clonal dissemination. Among all isolates harboring the *bla*_IMP-4_ carbapenemase gene, four isolates identified as predominant ST74, also contained *bla*_CMY−2_. One strain, designated as rare ST194, carried *bla*_CMY-1_. The *E. cloacae* strain, harboring both *bla*_IMP-4_ and *bla*_CMY-1_, was resistant to all three tested carbapenems. The *bla*_IMP-4_ gene was located on a highly mobile class 1 integron, showing a new form of the *bla*_IMP-4_-*qacG*-*aacA4* array. This is the first description of IMP-4-producing *E. cloacae* strains in Korea. This observation implicates the widespread of *bla*_IMP-4_ in *Enterobacteriaceae* clinical isolates and provides insights into the epidemic potential and clinical therapeutic importance of IMP-4-producing *E. cloacae* for healthcare-associated infections.

## Introduction

**Figure 1:**
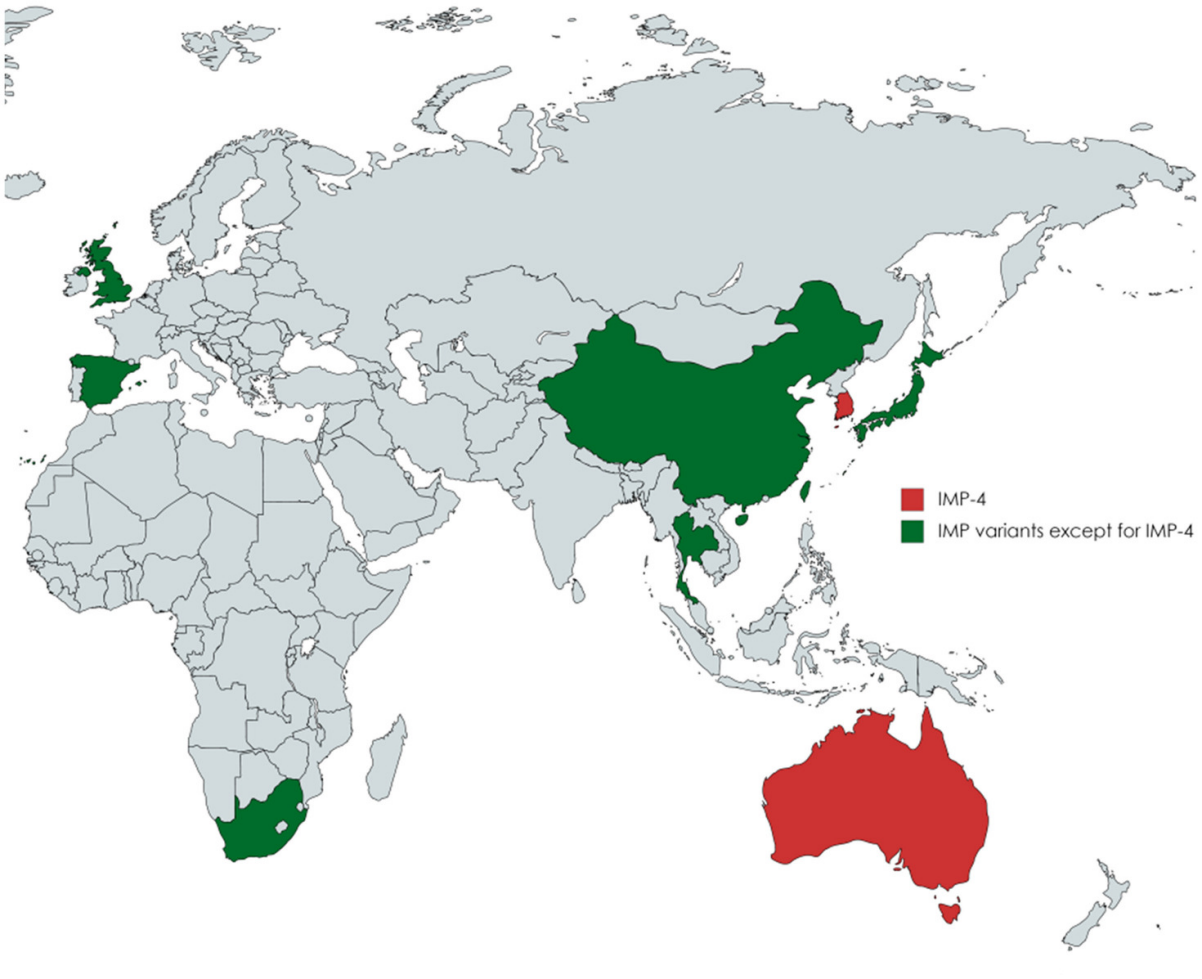
A map showing the global distribution of IMP-producing *Enterobacter cloacae* isolates. IMP-4-producing *E. cloacae* strains were especially described in Australia and Korea. IMP-type except for IMP-4 *E. cloacae* isolates have been reported in Taiwan (IMP-8), China (IMP-1 and IMP-34), Thailand (IMP-14), Japan (IMP-1 and IMP-11), Spain (IMP-13), United Kingdom (IMP-1), and South Africa.

The spread of carbapenemase-producing *Enterobacteriaceae* (CPE) has become a prominent health-care challenge worldwide in the treatment of infectious diseases. Carbapenemases, including *Klebsiella pneumoniae* carbapenemase (KPC), imipenemase (IMP), New Delhi metallo-β-lactamase (NDM), Verona integron-encoded metallo-β-lactamase (VIM), and oxacillinase (OXA)-48 medicated antibiotic resistance (Nordmann et al., 2011; Shi et al., 2017). IMP-type CPEs have been reported globally (Queenan and Bush, 2007; Tzouvelekis et al., 2012) and have become the predominant form in Australia (Espedido et al., 2008; Leung et al., 2013; Sidjabat et al., 2015) since the first report of IMP-1 from *Pseudomonas aeruginosa* in Japan (Watanabe et al., 1991). One of the most commonly observed IMP variants was IMP-4 in clinical *Enterobacteriaceae* isolates (Leung et al., 2013; Hu et al., 2014), which was firstly detected in Hong Kong (Chu et al., 2001). Among more than 11 different species of IMP-4-producing CPE, *Enterobacter cloacae* has emerged as the predominant species (Sidjabat et al., 2015; Cao et al., 2017). IMP-type *E. cloacae* isolates have been found in Taiwan (IMP-8), China (IMP-1 and IMP-34), Thailand (IMP-14), Japan (IMP-1 and IMP-11), Spain (IMP-13), United Kingdom (IMP-1), and South Africa (Figure 1; Chen et al., 2009; Shet et al., 2011; Hayakawa et al., 2014; Wang et al., 2015; Osei Sekyere, 2016; Matsumura et al., 2017). IMP-4-producing *E. cloacae* was particularly reported in Australia and caused clinical outbreaks, which brought greater challenges to infection control (Leung et al., 2013; Chapuis et al., 2016; Pang et al., 2016). The highly mobile class 1 integron facilitates global spread of the *bla*_IMP-4_ gene (Espedido et al., 2008; Partridge et al., 2012).

Until now, carbapenem-resistant *E. cloacae* has rarely been reported in Korea since the initial VIM-2-producing isolate in 2003 (Jeong et al., 2003). Here, we described the clinical characteristics and molecular mechanisms of the first IMP-4-producing *E. cloacae* clinical isolates in Korea.

## Materials and methods

### Bacterial strains

A total of 792 *E. cloacae* clinical isolates have been identified at a university hospital in Korea between March 2014 and February 2016. Among the isolates, five carbapenemase-producing *E. cloacae* strains (0.6%), YUMC1, YUMC2, YUMC3, YUMC4, and YUMC5 were included in this study. The isolates were identified as *E. cloacae* using the Vitek GNI card (bioMérieux, Marcy l'Étoile, France) and 16S rRNA sequencing (Lane et al., 1985; Mao et al., 2012; Mezzatesta et al., 2012; Jeong et al., 2015). This study was carried out in accordance with the recommendations of Institutional Review Board of Kosin University Gospel Hospital, Busan, Korea; with written informed consent from all subjects. All subjects gave written informed consent in accordance with the Declaration of Helsinki. We primarily focused on the analysis of the isolated strains and made our effort to anonymize private information of infected patients.

### Antimicrobial susceptibility testing

Antimicrobial susceptibilities were determined by the Vitek card AST-N246 (bioMérieux). Carbapenem producers were identified by modified Hodge test on MacConkey agar (Becton, Dickinson and Company, Sparks, MD, USA). The performance of modified Hodge test was reported to be better with MacConkey agar, containing bile compounds, than with Mueller-Hinton agar for screening carbapenemase-producing Gram-negative bacilli (K. Lee et al., 2010). Carbapenemase production was confirmed by KPC+MBL Confirm ID Kit (Rosco Diagnostica, Taastrup, Denmark) using tablets containing meropenem (10 μg) alone or supplemented with dipicolinic acid (1,000 μg), phenylboronic acid (400 μg), and cloxacillin (750 μg), and Mueller-Hinton agar (Oxoid Ltd., Basingstoke, UK). The minimum inhibitory concentrations (MICs) for imipenem, meropenem, and ertapenem were determined using E-test strips (AB Biodisk, Solna, Sweden). The breakpoints were applied according to the Clinical and Laboratory Standards Institute (CLSI) guidelines (Clinical Laboratory Standards Institute, 2016). Double-disk synergy test (DDST) for the detection of extended-spectrum β-lactamases (ESBLs) was also performed according to the CLSI guideline.

### Pulsed-field gel electrophoresis

Pulsed-field gel electrophoresis (PFGE) was performed to confirm the clonality of the IMP-4-producing *E. cloacae* isolates. *Xba*I (Roche, Mannheim, Germany)-digested genomic DNA was prepared at 37°C for 12–14 h. DNA fragments were separated using a CHEF-DRII System (Bio-Rad, Hercules, CA, USA). Banding patterns were analyzed with InforQuestFP software version 4.5 (Bio-Rad) to generate a dendrogram.

### Multilocus sequence typing

Multilocus sequence typing (MLST) for seven housekeeping genes, including *dnaA, fusA, gyrB, leuS, pyrG, rplB*, and *rpoB*, was conducted. After PCR and sequencing, nucleotide sequences were compared with those in the MLST database (http://pubmlst.org/ecloacae) to identify allelic numbers and sequence types (ST).

### Polymerase chain reaction and sequencing

The genomic DNA of five isolates were extracted via the boiling lysis method (L. Chen et al., 2011). The genes for 16S rRNA, carbapenemase, integron components, fluoroquinolones, ESBLs, and plasmid-mediated AmpCs were amplified by polymerase chain reaction (PCR) and sequenced using the primers (Lane et al., 1985; Jeong et al., 2003; Bae et al., 2007, 2011; Mao et al., 2012; Hong et al., 2015) described in Table 1. Briefly, the PCR program was as follows: 94°C denaturation for 5 min, followed by 30 cycles of 94°C denaturation for 30 s, then 55–60°C annealing for 30 s, and subsequently 72°C extension for 30 s, followed by 72°C final extension for 7 min. The amplified products were sequenced and the nucleotide sequences were compared by the Basic Local Alignment Search Tool (BLAST) (https://www.ncbi.nlm.nih.gov/BLAST) (Jeong et al., 2015). Genetic organization of class 1 integron carrying the *bla*_IMP-4_ gene cassette of a plasmid was investigated by PCR mapping and sequencing of the regions surrounding the gene using the primers described in Table 1 (Jeong et al., 2003; Bae et al., 2007; Hong et al., 2015). The integron variant was identified using INTEGRALL database (http://integrall.bio.ua.pt/) (Moura et al., 2009).

**Table 1:** Nucleotide sequences of primers used for the identification of species, the detection of resistant genes, and genetic environments in this study.

**Class^a^**	**Target gene(s) or region**	**Primer name**	**Sequence (5′ to 3′)**	**References**	**Position in Figure 2**
Identification	16S rRNA	16S-F	AGAGTTTGATYMTGGCTCAG	Mao et al., 2012	
		16S-R	CCGTCAATTCMTTTRAGTTT	Lane et al., 1985	
Carbapenemase	*bla*_IMP_ cluster	10IMP-F	AAGGCGTTTATGTTCATACTTCG	Hong et al., 2015	1
		IMP-bF	TGGTAAGGCAAAACTGGTTG	This study	5
		IMP-mR	TGATGAAGGCGTTTATGTTCA	This study	4
		10IMP-R	TTTAACCGCCTGCTCTAATGTAA	Hong et al., 2015	2
QAC	*qacG*	qacG-F	GGTTATTTCTGGCTACGTCCA	This study	7
		qacG-R	AGCAAGTTGAGCACAGCAAC	This study	6
Integron CS	*IntI1*	5CS	CTTCTAGAAAACCGAGGATGC	Jeong et al., 2003	3
	*sul1*	sul1-R	GGGTTTCCGAGAAGGTGATT	Bae et al., 2007	10
Fluoroquinolones	*aac(6′)-Ib-cr*	aac(6′)-Ib-F	TGACCTTGCGATGCTCTATG	This study	9
		aac(6′)-Ib-R	TTAGGCATCACTGCGTGTTC	This study	8
	*qnrA*	qnrAa-F	GAACCAACCCCATGTTTGC	This study	
		qnrAa-R	AGTCCCGACCAGACTGCATA	This study	
	*qnrB1*	qnrB1-F	ACCTGAGCGGCACTGAATTTA	This study	
		qnrB1-R	TCGCAATGTGTGAAGTTTGC	This study	
	*qnrB4*	qnrB4-F	GATGACTCTGGCGTTAGTTGC	This study	
		qnrB4-R	CCATGACAGCGATACCAAGA	This study	
	*qnrD*	qnrD-F	CGAGATCAATTTACGGGGGAAT	This study	
		qnrD-R	TCGGTGAACAATAACACCTAAAC	This study	
	*qnrS1*	qnrS-F	GACGTCCTAACTTGCGTGAT	This study	
		qnrS-R	ACTTTAGTCTGACTCTTTCAGTGATGC	This study	
ESBLs; Ambler class A	*bla*_TEM_ cluster	TEM-F	TCCGCTCATGAGACAATAACC	Bae et al., 2011	
		TEM-R	ACGCTCAGTGGAACGAAAAC	Bae et al., 2011	
	*bla*_SHV_ cluster	SHV-F	CGCCGGGTTATTCTTATTTG	Bae et al., 2011	
		SHV-R	CCACGTTTATGGCGTTACCT	Bae et al., 2011	
	*bla*_VEB_ cluster	VEB-F	AAAATGCCAGAATAGGAGTAGCA	Bae et al., 2011	
		VEB-R	TCCACGTTATTTTTGCAATGTC	Bae et al., 2011	
	*bla*_GES/IBC_ cluster	GES-F	CGCTTCATTCACGCACTATT	Bae et al., 2011	
		GES-R	GTCCGTGCTCAGGATGAGTT	Bae et al., 2011	
	*bla*_CTX−M−1_ cluster	CMT-M-1-F	CCGTCACGCTGTTGTTAGG	Bae et al., 2011	
		CMT-M-1-R	ACGGCTTTCTGCCTTAGGTT	Bae et al., 2011	
	*bla*_CTX−M−9_ cluster	CMT-M9-F	CAAAGAGAGTGCAACGGATG	Bae et al., 2011	
		CMT-M9-R	CCTTCGGCGATGATTCTC	Bae et al., 2011	
	*bla*_KPC_ cluster	KPC-F	GTCACTGTATCGCCGTCTAGT	Hong et al., 2015	
		KPC-R	TGGTGGGCCAATAGATGATT	Hong et al., 2015	
	*bla*_NMC−A/IMI_ cluster	IMC-F	CATTTTTCTCACAGGCCAATAC	This study	
		IMC-R	TGCTTGGCTTCTTTTTCGTT	This study	
Ambler class B	*bla*_VIM_ cluster	VIM-2F	ATCATGGCTATTGCGAGTCC	Hong et al., 2015	
		VIM-2R	ACGACTGAGCGATTTGTGTG	Hong et al., 2015	
Ambler class C; AmpCs	*bla*_CMY-1_ cluster	CMY-1F	GTCAGCGAGCAGACSCTGTT	This study	
		CMY-1R	TAGTTGCGRTTGGCCAGC	This study	
	*bla*_CMY−2_ cluster	CMY-2F	GCAGGCYATTCCGGGTATG	This study	
		CMY-2R	GCYACGTAGCTGCCAAAYCC	This study	
Ambler class D	*bla*_OXA−48_ cluster	OXA48-F	CAGCAAGCATTTACCAATAAT	This study	
		OXA48-R	GGCATATCCATATTCATCGC	This study	

a *QAC, quaternary ammonium compounds; CS, Conserved segment; ESBLs, extended-spectrum β-lactamases*.

### Nucleotide sequence accession number

Nucleotide sequence data for YUMC2 are available under the GenBank accession number KY884003 and assigned In1456 for class 1 integron based on the INTEGRALL database (http://integrall.bio.ua.pt/) (Moura et al., 2009).

## Results

### Description of the patients

The clinical characteristics of the patients infected with five isolates are summarized in Table 2. The carbapenemase-producing *E. cloacae* strains were isolated from various departments and two of them were recovered from the open wounds in diabetic feet. Most of the patients suffered from underlying diseases such as hypertension, diabetes mellitus and/or cancer causing immunocompromised conditions.

**Table 2:** Clinical characteristics of the patients infected with IMP-4-producing *E. cloacae* isolates.

**Strain**	**Sex/Age**	**Department**	**Specimen**	**Date of isolation (year/month)**	**Diagnosis**	**Comorbidity**
YUMC1	M/44	OS	Wound	2014/9	Open wound on right Toe; Diabetes mellitus foot necrosis	Hypertension; Type 2 diabetes mellitus
YUMC2	M/47	PS	Wound	2015/2	Open wound on right foot	Hypertension; Type 2 diabetes mellitus; Old cerebrovascular attack
YUMC3	F/41	GS	Ascitic fluid	2015/9	Invasive carcinoma of right breast	Renal cell carcinoma; Chronic gastritis
YUMC4	F/70	NS	Urine	2016/2	Spontaneous SAH with right PICA aneurysm	Hypertension; Cerebral infarction
YUMC5	F/20	OBGY	Vaginal swab	2016/2	Vaginitis	Not specified

*OS, orthopedic surgery; PS, plastic surgery; GS, general surgery; NS, neurosurgery; OBGY, obstetrics gynecology; SAH, subarachnoid hemorrhage; PICA, posterior inferior cerebellar artery*.

### Antimicrobial susceptibility profiles

The antimicrobial susceptibility profiles of five *E. cloacae* isolates with *bla*_IMP-4_ are presented in Supplementary Table 1. All five isolates were not susceptible to ampicillin, amoxicillin-clavulanic acid, cephalosporins, and carbapenems, whereas susceptible to amikacin, gentamicin, tigecycline, and ciprofloxacin, except for YUMC2. DDST for ESBL was negative for all five isolates. The antimicrobial susceptibility profiles of the other 787 *E. cloacae* strains are also summarized in Supplementary Table 2. The overall patterns are similar to those of five IMP-4-producing isolates, except for the carbapenems.

### Resistance to carbapenems

All five isolates were positive as carbapenem producers in the modified Hodge test and KPC+MBL Confirm ID Kit (Rosco Diagnostica). The MICs were determined using E-test strips (AB Biodisk) and the results for imipenem, meropenem, and ertapenem are presented in Table 3. All isolates were not susceptible to at least one of the carbapenems using CLSI breakpoints. Notably, YUMC2 was resistant to all tested carbapenems and had higher MICs than other isolates.

**Table 3:** Pulsed-field gel electrophoresis (PFGE)-based dendrogram and multilocus sequence typing (MLST) of IMP-4-producing *E. cloacae* isolates^a^.

**% Similarity index**			**PFGE-Xbal pattern**	**Isolates**	**MIC(μg/ml) of**^b^			**β-lactamases**	**MLST**							
**90**	**95**	**100**			**IPM**	**MEM**	**EPM**		**Sequence type**	***dna A***	***fus A***	***gyr B***	***leu S***	***pyr G***	***rpt B***	***rpo B***
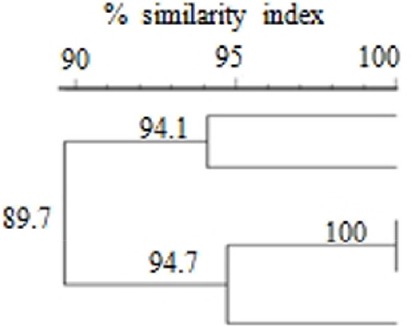			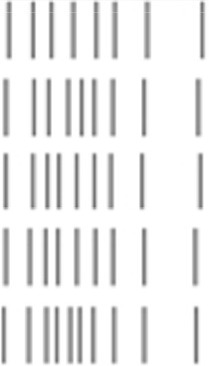	YUMC 2	4	4	4	IMP-4, CMY-1	74	8	33	6	9	9	6	8
				YUMC 3	1	1	1	IMP-4, CMY-2	194	11	6	4	13	39	4	9
				YUMC 4	2	4	4	IMP-4, CMY-2	194	11	6	4	13	39	4	9
				YUMC 5	1	1	1	IMP-4, CMY-2	194	11	6	4	13	39	4	9
				YUMC 1	2	<0.5	0.5	IMP-4, CMY-2	194	11	6	4	13	39	4	9

a *Similarity index scale is shown above the dendrogram, and % similarity indexes are indicated over the nodes*.

b *The MIC values of ≤ 1, 2, and ≥4 are susceptible, intermediate, resistant to imipenem and meropenem, respectively. The breakpoints for ertapenem are ≤ 0.5, 1, and ≥1 according to the interpretative criteria of Clinical and Laboratory Standards Institute (CLSI) guideline. MIC, minimum inhibitory concentration; IPM, imipenem; MEM, meropenem; EPM, ertapenem*.

### Clonality of the isolates

YUMC4 and YUMC5 strains presented identical PFGE patterns and the other isolates also showed highly similar patterns based on the criteria of 85% similarity (Table 3). The strains, isolated same years, presented close relationship.

### Sequence type

The MLST assay assigned the isolates to two STs (Table 3). YUMC2 was assigned to predominant ST74. Four out of the five IMP-4-producing *E. cloacae* strains were rare ST194, showing significant clonal similarity.

### Carbapenemase genes and genetic environment

PCR screening demonstrated the presence of *bla*_IMP-4_ in all *E. cloacae* isolates (Table 3). In addition, YUMC2 was also positive for CMY-1. The other strains contained IMP-4 and CMY-2 simultaneously. The ESBL genes were not detected whereas, *aac(6*′*)-Ib-cr* and *qnrS1* relevant to fluoroquinolones were found. In this study, *bla*_CMY-1_, *bla*_CMY−2_, *bla*_IMP−4_, *aac(6)-Ib-cr*, and *qnrS1* were identical to previously reported sequences deposited in GenBank database under accession numbers X92508.1, X91840.1, AF244145.1, CP023487.1, and AB187515.1, respectively.

**Figure 2:**
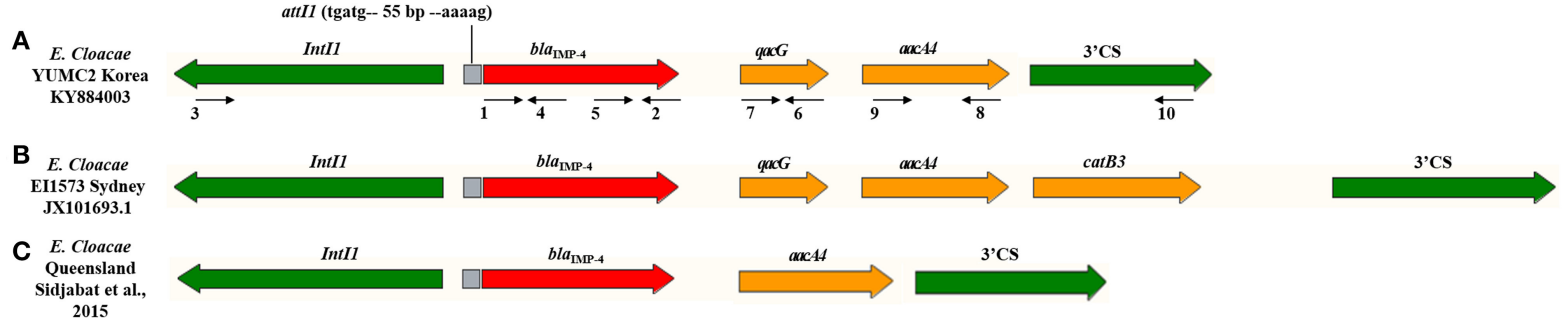
Schematic representation of the class 1 integron gene cassettes bearing the *bla*_IMP-4_ genes in *E. cloacae* isolates. Genes and their directions of transcription are described as broad arrows. The gray box indicates recombination site. The primers, detailed in Table 1, for PCR mapping are depicted as narrow arrows with numbers. The red arrow of *bla*_IMP-4_ is related to carbapenemase. The yellow arrow of *qacG* and *aacA4* are associated with resistance to quaternary ammonium compounds and fluoroquinolone, respectively. The 5′ conserved segment (CS) of *IntI1* and 3′ CS of *sul1* are presented with green arrow. **(A)** *E. cloacae* YUMC2 in this study with Genbank accession no. KY884003. **(B)** *E. cloacae* EI1573 from Sydney, Australia with Genbank accession no. JX101693.1. **(C)** *E. cloacae* from Queensland, Australia reported by Sidjabat et al. (2015).

PCR mapping and sequencing generated a 3,585-bp segment that shared 99% identity with *E. cloacae* pEI1573 (GenBank accession no. JX101693.1) (Partridge et al., 2012). The *bla*_IMP-4_-gene was located on class 1 integron In1456, consisted of novel *bla*_IMP-4_-*qacG2*-*aacA4* cassette array (Figure 2).

## Discussion

*E. cloacae* is frequently implicated in serious nosocomial infections with high mortality. Majority of patients were reported to be immunocompromised, similar to our patients (Qureshi et al., 2011). Clinical outbreaks of *E. cloacae* in the hematology ward, burns unit, and intensive care unit have persisted, despite of concerted infection control to prevent ongoing transmission (Leung et al., 2013; Chapuis et al., 2016; Pang et al., 2016). VIM-2, NDM-1, and IMP-1, frequently found in Asia, have been previously reported mechanisms of carbapenem-resistant *E. cloacae* in Korea (Jeong et al., 2003; Kim et al., 2015; Lee et al., 2017). Meanwhile, IMP-4-producing *E. cloacae* isolates have been mainly found in Australia (Peleg et al., 2005; Leung et al., 2013). The first detection of IMP-4 in this study implicates that the plasmid-mediated *bla*_IMP-4_ eventually spread in *E. cloacae* clinical isolates in Korea.

IMP-4 was reported to be strongly active against imipenem and meropenem, with 0.25–16 MIC range (Chu et al., 2001). The MICs of our isolates showed that all five strains were not susceptible to at least one of the carbapenems, including imipenem, meropenem, and ertapenem. Antibiotic resistance profiles of *bla*_IMP_-positive *Enterobacteriaceae* isolates showed 25% resistance, 57% intermediate resistance, and 18% susceptibility to meropenem and 6% resistance, 33% intermediate resistance, and 61% susceptibility to imipenem in a previous study (Dolejska et al., 2016). Natural antibiotic susceptibility of *E. cloacae* complex to carbapenems were reported to be susceptible (Stock et al., 2001), however, the presence of IMP-4 would influence on the antibiotic profiles.

The antimicrobial susceptibility profiles of *E. cloacae* isolates in this study were similar to the intrinsic patterns of antibiotics (Mezzatesta et al., 2012). However, 5 strains containing *bla*_IMP-4_ were not susceptible to carbapenems and YUMC2 was resistant to ciprofloxacin. The detected genes, *aac(6*′*)-Ib-cr* and *qnrS1* relevant to fluoroquinolones might be associated with this results. Nevertheless, the *cr* variant of *aac(6*′*)-Ib* confers reduced susceptibility to ciprofloxacin by *N*-acetylation of its piperazinyl amine (Robicsek et al., 2006), ciprofloxacin resistance was not related to *aac(6*′*)-Ib-cr* prevalence (Park et al., 2006). Interestingly, the isolates co-carrying *aac(6*′*)-Ib-cr* and *qnrS1* were also reported to be sensitive to quinolones (Huang et al., 2012). Therefore, these genes seems to supplement other quinolone resistance mechanisms rather than confer directly to resistance. Although, the *aac(6*′*)-Ib-cr* and *qnrS1* genes were frequently found to be co-carried with various ESBLs, becoming therapeutic threats (Huang et al., 2012; Mezzatesta et al., 2012), our isolates harbored *bla*_IMP-4_ without ESBLs.

The homogeneity of five strains was analyzed using PFGE. Although the strains were isolated from various clinical departments, the high similarity of PFGE patterns of isolates, especially in the same years, might be the evidence of hospital-wide clonal dissemination.

According to MLST results, YUMC2 was designated to ST74, the most predominant clonal lineage with increased epidemic potential based on previous *E. cloacae* clonality studies (Fernández et al., 2015; Guillard et al., 2015; Izdebski et al., 2015). *E. cloacae* ST74 had higher carbapenems MICs than other isolates, similar to the results of previous studies, and was assumed to confer with the spread of the resistance to carbapenems (Guillard et al., 2015; Izdebski et al., 2015). The other four IMP-4-producing *E. cloacae* strains were ST194, presenting significant genetic similarity. To the best of our knowledge, available studies for *E. cloacae* ST194 were rare, indicating that this is the first report of clinical *E. cloacae* ST194.

PCR results showed the presence of CMY-1 in YUMC2 and CMY-2 in the other strains as well as IMP-4. Prior studies demonstrated that the most frequently reported AmpC β-lactamase was CMY, consisting of 92.7% of CMY-2 among *Enterobacteriaceae* isolates in the Asia-Pacific region (Sheng et al., 2013). The combination of *bla*_IMP-4_ and *bla*_CMY−2−like_ was found from one clinical *E. cloacae* isolate among the CPE in Australia (Sidjabat et al., 2015). In addition, the coexistence of *bla*_IMP-4_ and *bla*_CMY-1_ in *E. cloacae* strain was not reported previously and this is the first description of *E. cloacae*, coproducing IMP-4 and CMY-1 with resistance to all three carbapenems.

When comparing the product of sequencing of our study to *E. cloacae* pEI1573 (GenBank accession no. JX101693.1) (Partridge et al., 2012), both of the *bla*_IMP-4_ genes of our study and pEI1573 were located on class 1 integrons. However, the gene cassettes compositions were slightly different between YUMC2 and pEI1573, containing a reference sequence of typical Australian class 1 integron array (Figure 2). The *bla*_IMP-4_-*qacG*-*aacA4*-*catB3* cassette array of pEI1573 from Sydney, Australia is almost identical to those of pJIBE401 from Sydney index isolate *K. pneumoniae* (GenBank accession no. AJ609296) (Espedido et al., 2005), pCTX-M3 from *Citrobacter freundii* in Poland (GenBank accession no. AF550415) (Gołebiewski et al., 2007), and pCTX-M360 from *K. pneumoniae* in China (GenBank accession no. EU938349) (Zhu et al., 2009). Meanwhile, the class 1 integron of our study consisted of *bla*_IMP-4_-*qacG*-*aacA4* and a different array form composed of *bla*_IMP-4_-*aacA4*, which was reported previously from Queensland, Australia (Sidjabat et al., 2015). These cassette arrays, found in diverse isolates with slightly different genetic contexts, suggest movement of the array by homologous recombination and the worldwide dissemination potential of *bla*_IMP-4_ gene.

In the respect of epidemiological relationship, the class 1 integrons of Australia and Korea, containing *bla*_IMP-4_ genes of *E. cloacae* isolates, revealed similar gene cassettes, except for *catB3* or *qacG*. Geographically, Australia and Korea are located at the rim of Asian-pacific region. Further, a large-scale transmission of *bla*_IMP-4_ of *E. cloacae* isolates, predominant from of CPE in Australia (Sidjabat et al., 2015), through silver gulls of Australia was previously reported (Dolejska et al., 2016).

In conclusion, we report the first IMP-4-producing *E. cloacae* strains identified as predominant ST74 and rare ST194 in Korea. Furthermore, it is the first description of *bla*_IMP-4_ and *bla*_CMY-1_ coexistence and a new class 1 integron cassette array form in *Enterobacteriaceae*. This finding implicates the emergence of plasmid-mediated *bla*_IMP-4_ on the highly mobile class 1 integron in *Enterobacteriaceae* clinical isolates in Korea with great concern for widespread and therapeutic threats. In addition, it provides insights into the epidemic potential and clinical importance of IMP-4-producing *E. cloacae* for hospital-acquired infections.

## Author contributions

SJ: analyzed the data, and wrote the manuscript; IKB: designed and performed the experiments, and revised the manuscript; JHL and CHL: helped the experiments and the writing of the manuscript.

### Conflict of interest statement

The authors declare that the research was conducted in the absence of any commercial or financial relationships that could be construed as a potential conflict of interest.
