# Supplementary material for: Molecular Characteristics of First IMP-4-Producing Enterobacter cloacae Sequence Type 74 and 194 in Korea
Source: Front Microbiol. 2017 Nov 28;8:2343. doi: 10.3389/fmicb.2017.02343 (PMC5741837; doi:10.3389/fmicb.2017.02343)
Supplement: Supplementary file 1 [file Table1.doc]

Supplementary Material

# Molecular Characteristics of First IMP-4-Producing *Enterobacter cloacae* Sequence Type 74 and 194 in Korea

Jong Ho Lee^1†^, Il Kwon Bae^2†^, Chae Hoon Lee^1^, Seri Jeong^3*^

*** Correspondence:** Seri Jeong: [hehebox@naver.com](mailto:hehebox@naver.com)

## Supplementary Table 1 | Antimicrobial susceptibility profiles of five IMP-4-producing *E. cloacae* isolates^a^.

| Antibiotics | YUMC1 | YUMC2 | YUMC3 | YUMC4 | YUMC5 |
| --- | --- | --- | --- | --- | --- |
| Ampicillin | ≥ 32, R | ≥ 32, R | ≥ 32, R | ≥ 32, R | ≥ 32, R |
| Amoxicillin-clavulanic acid | ≥ 32, R | ≥ 32, R | ≥ 32, R | ≥ 32, R | ≥ 32, R |
| Piperacillin-tazobactam | ≥ 128, R | 32, I | 16, S | ≥ 128, R | 64, I |
| Cefazolin | ≥ 64, R | ≥ 64, R | ≥ 64, R | ≥ 64, R | ≥ 64, R |
| Cefoxitin | ≥ 64, R | ≥ 64, R | ≥ 64, R | ≥ 64, R | ≥ 64, R |
| Cefotaxime | ≥ 64, R | ≥ 64, R | ≥ 64, R | ≥ 64, R | ≥ 64, R |
| Ceftazidime | ≥ 64, R | ≥ 64, R | ≥ 64, R | ≥ 64, R | ≥ 64, R |
| Cefepime | 4 , SDD | 8, SDD | 4, SDD | ≥ 64, R | 4, SDD |
| Aztreonam | ≤ 1, S | ≤ 1, S | ≤ 1, S | ≥ 64, R | ≤ 1, S |
| Ertapenem | ≥ 8, R | ≥ 8, R | 4, R | ≥ 8, R | 4, R |
| Imipenem | ≥ 16, R | ≥ 16, R | ≥ 16, R | ≥ 16, R | ≥ 16, R |
| Amikacin | ≤ 2, S | 8, S | ≤ 2, S | ≤ 2, S | ≤ 2, S |
| Gentamicin | ≤ 1, S | ≤ 1, S | ≤ 1, S | ≤ 1, S | 2, S |
| Ciprofloxacin | 1, S | ≥ 4, R | 1, S | 1, S | 1, S |
| Tigecycline | 2, S | 2, S | 2, S | 2, S | 2, S |
| Trimethoprim-sulfamethoxazole | ≤ 20, S | ≥ 320, R | ≤ 20, S | ≤ 20, S | ≤ 20, S |

^a^ Data are presented as minimum inhibitory concentration (mg/L), interpretation; The breakpoints were applied according to the Clinical and Laboratory Standards Institute (CLSI) guideline; R, resistant; S, susceptible; SDD, susceptible dose-dependent.
