# Supplementary material for: Molecular Characteristics of First IMP-4-Producing Enterobacter cloacae Sequence Type 74 and 194 in Korea
Source: Front Microbiol. 2017 Nov 28;8:2343. doi: 10.3389/fmicb.2017.02343 (PMC5741837; doi:10.3389/fmicb.2017.02343)
Supplement: Supplementary file 2 [file Table2.DOC]

Supplementary Material

# Molecular Characteristics of First IMP-4-Producing *Enterobacter cloacae* Sequence Type 74 and 194 in Korea

Jong Ho Lee^1†^, Il Kwon Bae^2†^, Chae Hoon Lee^1^, Seri Jeong^3*^

*** Correspondence:** Seri Jeong: [hehebox@naver.com](mailto:hehebox@naver.com)

## Supplementary Table 2 | Antimicrobial susceptibility profiles of the other 787 *E. cloacae* strains without *bla*_IMP-4_^a^.

| Antibiotics | R | I | S |
| --- | --- | --- | --- |
| Ampicillin | 762 (96.8) | 1 (0.1) | 24 (3.0) |
| Amoxicillin-clavulanic acid | 763 (97.0) | 0 (0.0) | 24 (3.0) |
| Piperacillin-tazobactam | 161 (20.5) | 48 (6.1) | 578 (73.4) |
| Cefazolin | 762 (96.8) | 0 (0.0) | 25 (3.2) |
| Cefoxitin | 767 (97.5) | 0 (0.0) | 20 (2.5) |
| Cefotaxime | 281 (35.7) | 4 (0.5) | 502 (63.8) |
| Ceftazidime | 251 (31.9) | 9 (1.1) | 527 (67.0) |
| Cefepime | 35 (4.4) | 6 (0.8) | 746 (94.8) |
| Aztreonam | 257 (32.7) | 5 (0.6) | 525 (66.7) |
| Ertapenem | 103 (13.1) | 47 (6.0) | 637 (80.9) |
| Imipenem | 19 (2.4) | 38 (4.8) | 730 (92.8) |
| Amikacin | 6 (0.8) | 2 (0.3) | 779 (99.0) |
| Gentamicin | 45 (5.7) | 11 (1.4) | 731 (92.9) |
| Ciprofloxacin | 105 (13.3) | 27 (3.4) | 655 (83.2) |
| Tigecycline | 25 (3.2) | 38 (4.8) | 724 (92.0) |
| Trimethoprim-sulfamethoxazole | 131 (16.6) | 0 (0.0) | 656 (83.4) |

^a^ Data are expressed as number of strains (%); The breakpoints were applied according to the Clinical and Laboratory Standards Institute (CLSI) guideline; R, resistant; I, intermediate; S, susceptible.
